# Fusing simulation and experiment: The effect of mutations on the structure and activity of the influenza fusion peptide

**DOI:** 10.1038/srep28099

**Published:** 2016-06-15

**Authors:** Diana Lousa, Antónia R. T. Pinto, Bruno L. Victor, Alessandro Laio, Ana S. Veiga, Miguel A. R. B. Castanho, Cláudio M. Soares

**Affiliations:** 1 ITQB, Instituto de Tecnologia Química e Biológica António Xavier, Universidade Nova de Lisboa, Av. da República, 2780-157 Oeiras, Portugal; 2 Instituto de Medicina Molecular, Faculdade de Medicina da Universidade de Lisboa, Av. Professor Egas Moniz, 1649-028 Lisboa, Portugal; 3 SISSA/ISAS, Statistical and biological physics, Via Beirut 2-4 Trieste, Italy

## Abstract

During the infection process, the influenza fusion peptide (FP) inserts into the host membrane, playing a crucial role in the fusion process between the viral and host membranes. In this work we used a combination of simulation and experimental techniques to analyse the molecular details of this process, which are largely unknown. Although the FP structure has been obtained by NMR in detergent micelles, there is no atomic structure information in membranes. To answer this question, we performed bias-exchange metadynamics (BE-META) simulations, which showed that the lowest energy states of the membrane-inserted FP correspond to helical-hairpin conformations similar to that observed in micelles. BE-META simulations of the G1V, W14A, G12A/G13A and G4A/G8A/G16A/G20A mutants revealed that all the mutations affect the peptide’s free energy landscape. A FRET-based analysis showed that all the mutants had a reduced fusogenic activity relative to the WT, in particular the mutants G12A/G13A and G4A/G8A/G16A/G20A. According to our results, one of the major causes of the lower activity of these mutants is their lower membrane affinity, which results in a lower concentration of peptide in the bilayer. These findings contribute to a better understanding of the influenza fusion process and open new routes for future studies.

Influenza virus is a devastating human pathogen, causing hundreds of thousands of deaths every year, which rise to millions in pandemic years (http://www.who.int/mediacentre/factsheets/2003/fs211/en). One of the most important steps in the infection process of this virus is the fusion between the viral and host membranes. This process is mediated by the glycoprotein hemagglutinin (HA), a homotrimer in which each monomer is composed by two polypeptide chains (HA1 and HA2), linked by a disulfide bridge^1^. The HA1 subunit recognizes and binds to the host cell receptors, located on the host membrane. The virus is then engulfed by endocytosis and the low pH of the late endosome triggers a large conformational change on HA, in which HA1 detaches from HA2 and the latter subunit becomes extended. At this point, the N-terminal region of HA2, termed fusion peptide (FP), inserts into the host membrane, promoting fusion between the viral and host lipid bilayers^1^. Although it is clear that the FP plays a crucial role in the fusion process, the mechanism of action of this peptide remains elusive. Proposed mechanisms include destabilization of the membrane by inducing local disorder, increasing membrane curvature, and promoting sinking of the lipid headgroups and protrusion of lipid tails^2^,^3^,^4^,^5^,^6^,^7^.

The fusion peptide comprises the first ~23 amino acid residues of HA2 and this segment is able to induce lipid mixing of liposomes (hemifusion), even in the absence of the rest of the protein^8^,^9^. The structure adopted by the FP in the host membrane is thought to be determinant for its function and several studies have tried to address this question^10^,^11^,^12^,^13^,^14^,^15^. Given the difficulties associated with obtaining peptide structures in membrane bilayers, the most common strategy was to solve the NMR structure of the FP in detergent micelles, in an attempt to mimic the membrane environment. These studies showed that the influenza FP adopts a helix-turn-helix conformation. The angle between the two helices (kink angle) is affected by the peptide length: a FP segment comprising 20 amino acid residues adopts an inverted V-structure at the endosome pH^10^, whereas a 23-residue long peptide adopts a considerably more closed hairpin-like structure^13^. The closed structure of the 23-residue long peptide is stabilized by a glycine zipper formed by two pairs of glycine residues (G4-20, G8-16) and also by the interaction of the N and C-terminal regions of the peptide^13^. Solid state NMR experiments have also been used to probe the structure of the FP in a membrane bilayer^16^,^17^. However, although these studies provided interesting insights into the conformation of the peptide in the membrane, they could not provide an atomic resolution description of the peptide’s structure.

The influenza FP is highly conserved and mutations in different residues have been shown to decrease or abolish its fusogenic activity. A compilation of the effect of different mutations can be found in ref. 18. Studies with mutant HA proteins indicate that point mutations in the first residue (which is a glycine in the wild type protein) either inactivate or significantly impair the fusion activity (the only known exception is the G1A mutation)^19^,^20^. Mutations in the conserved residue W14, which is located in the kink region, have also been shown to abolish or significantly reduce the fusogenic activity of the influenza FP^18^,^21^. The substitution of G8 (which is part of the glycine zipper) by an alanine resulted in the loss of activity^19^, and the NMR structure of this mutant in DPC micelles revealed that it tends to adopt an open conformation^15^. This supports the notion that the close contact between glycine pairs located on the two helices (glycine zipper) is important to maintain the helical-hairpin structure, which seems to be determinant for the peptide’s function.

Molecular dynamics (MD) simulation studies have been used by many groups to analyze this intriguing system. The fusion of lipid vesicles and membranes has been simulated using both coarse-grain and atomistic models (for a recent review see ref. 22). Atomic-resolution simulations of vesicle fusion indicate that the process is triggered by the translocation of a few lipid tails from the fusing vesicles into the hydrophilic region^3^. The FP seems to induce the protrusion of the neighboring lipid tails, which may explain how the peptide promotes fusion^2^,^3^,^4^,^5^. Moreover, the FP attracts the lipid headgroups (which results in a decrease in the bilayer thickness in the surrounding region), and decreases the order of nearby lipids^2^,^5^,^23^,^24^,^25^,^26^. Coarse grain MD simulation studies indicate that the influenza FP promotes fusion by inducing lipidic phases with a large positive curvature^27^, or by stabilizing pores, which drives the elongation of the stalk^28^. Atomistic self-assembly simulations performed by our group, in which the membrane spontaneously grows around the peptide, thus avoiding the bias imposed by choosing the initial peptide location^2^, revealed that the peptide can adopt two different configurations: parallel to the membrane in the headgroup-lipid tail interface or a considerably more tilted membrane-spanning conformation. The latter orientation was observed in 4 out of 5 simulations, which suggests that it is more stable than the interfacial conformation. Moreover, when the peptide is in the tilted conformation it has a stronger effect on the membrane, lowering the bilayer thickness, disordering nearby lipids, and promoting lipid tail protrusion.

In short, although many experimental and theoretical studies have tried to unravel the structure of the FP and the mechanism by which it triggers the fusion process, a clear picture of the mechanism has not yet emerged, mainly due to the limitations of the methods that have been used. Experimentally, it is difficult to obtain structures of peptides in membranes. Simulation methods, such as standard MD, are often hampered by sampling problems, which are particularly severe in the viscous membrane environment.

In this work we used a combination of simulation and experimental techniques that allow at least partially circumventing these problems.

We performed bias-exchange metadynamics (BE-META) simulations^29^, which are substantially more efficient than standard MD^30^, and allow characterizing the free energy landscape of this peptide within excellent accuracy. This method was applied to the WT and four mutant peptides (G1V, W14A, G12A/G13A and G4A/G8A/G16A/G20A) in order to test the effect of these mutations. Our results show that the lowest free-energy conformation sampled by the WT peptide in the membrane corresponds to helical-hairpin structures similar to the one observed in detergent micelles. All the mutations we considered destabilize this conformation and the more pronounced effect is observed in the mutant G4A/G8A/G16A/G20A. The lowest free energy conformation of the mutants G1V and W14A is similar to that of the WT. However, these mutants also populate other conformations, which indicates that the helical-hairpin structure is not as stable as in the case of the WT.

The simulation results were complemented by a biophysical study using fluorescence spectroscopy to evaluate the interaction of the FP WT and mutants with membrane model systems, as well as their ability to induce lipid mixing. These analyses showed that all the mutant peptides were less efficient in promoting lipid mixing than the WT and the loss of efficiency is particularly severe for the mutants G12A/G13A and G4A/G8A/G16A/G20A. One of the major factors contributing to this activity decrease is the fact that the mutant peptides have a lower membrane affinity, which results in a lower membrane concentration. Additionally, secondary structure analysis, performed by Fourier Transform-Infrared Spectroscopy (FTIR) showed that the G12A/G13A and G4A/G8A/G16A/G20A tend to form β-sheet aggregates, which can also contribute to the loss of activity.

## Materials and Methods

The peptides used in this study were the WT FP (GLFGAIAGFIEGGWTGMIDGWYGS) and the mutants G1V (VLFGAIAGFIEGGWTGMIDGWYGS), W14A (GLFGAIAGFIEGGATGMIDGWYGS), G4A/G8A/G16A/G20A (GLFAAIAAFIEGGWTAMIDAWYGS), and G12A/G13A (GLFGAIAGFIEAAWTGMIDGWYGS). All the peptides were purchased with purity higher than 95% from JPT Peptide Technologies GmbH (Berlin, Germany). 1-palmitoyl-2-oleoyl-sn-glycero-3-phosphocholine (POPC), 1-palmitoyl-2-oleoyl-sn-glycero-3-phosphoethanolamine (POPE) and 1,2-dipalmitoyl-sn-glycero-3-phosphocholine (DPPC) were obtained from Avanti Polar Lipids (Alabaster, AL). Cholesterol, dipalmitoylphosphatidylehtanolamine-sulforhodamine B (RhB-PE) and 1,2-dihexadecanoyl-sn-glycero-3-phospho[N-4-nitrobenz- 2-oxa-1,3-diazolyl]ethanolamine (NBD-PE) were purchased from Sigma (St. Louis, MO). Acetate buffer (20 mM sodium acetate, 150 mM NaCl, pH 5) was used in all the measurements to mimic the endosomes environment. Peptides stock solutions were prepared by dissolving the peptides in dimethylsulfoxide (DMSO) before dilution with the buffer. The solubilization of all peptides was improved with mild bath sonication. Fluorescence spectroscopy measurements were conducted at room temperature in a Varian Cary Eclipse fluorescence spectrophotometer (Mulgrave, Australia). All the experimental assays were conducted in triplicate.

### Membrane interactions studies

Membrane partition studies were carried out with large unilamellar vesicles (LUV) composed of POPC/POPE 50:50 (mol %). LUV with ~100 nm diameter were obtained by extrusion techniques^31^. The studies were performed by adding small volumes of concentrated LUV stock solutions to the peptides samples (WT, G1V, G12A/G13A, G4A/G8A/G16A/G20A, at 16 μM, and W14A at 29 μM), with a 10 min incubation before measurements. Fluorescence emission spectra were scanned in the 300–450 nm range with an excitation wavelength of 280 nm. The fluorescence intensities were corrected for successive dilutions, background intensities and scatter. Partition curves were plotted, and the partition coefficient, *K*_*p*_, was determined as previously described^32^ to compare the affinity of the peptides.

### Lipid mixing

To study the lipid mixing in vesicles induced by the peptides, a Förster Resonance Energy Transfer (FRET)-based assay was used as previously described^33^. This assay is based on the decrease in resonance energy transfer between two membrane probes, RhB-PE and NBD-PE, when the lipids of the vesicles labeled with both probes are allowed to mix with lipids from unlabelled vesicles. The concentration of each of the fluorescent probes within the pre-fusion LUV membrane was 0.6 mol %. For this assay LUVs composed of POPC/POPE 50:50 (mol %) was used and prepared as described above. Labeled and unlabeled vesicles in a proportion of 1:4 were used at a total final lipid concentration of 100 μM. The fluorescence was measured with excitation at 470 nm and emission recorded between 500 and 650 nm. Phospholipid mixing was quantified on a percentage basis:


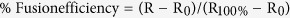


where R is the value of the ratio between the fluorescence intensity with emission at 530 nm and 588 nm, corresponding to the maximum fluorescence emission of NBD and RhB, respectively, obtained 10 min after the peptides addition (at a final concentration of 16 μM) to a mixture containing LUV having 0.6 mol % of each probe plus LUV without any fluorescent probe. R_0_ is the ratio before peptide addition (constant during the evaluated time range), and R_100%_ the ratio after addition of Triton X-100 at a final concentration of 1% (v/v).

### Secondary structure analysis

FTIR spectroscopy was used to analyze the peptides’ secondary structures. Attenuated total reflection infrared (ATR-FTIR) spectra were obtained on a Bruker Tensor27 Bio ATR II spectrophotometer (Ettlingen, Germany) equipped with a MCT detector (broad band 1200–420 cm^−1^, liquid N_2_ cooled) at a resolution of 4 cm^−1^. The spectrometer was continuously purged with dry air. The internal reflection element was a silicone (Si) ATR plate. Peptide samples (at 0.5 mg/mL) in the absence and presence of POPC LUV (2 mg/mL) were prepared in buffer, spread on the Si plate and dried until solvent evaporation. POPC LUVs were prepared as described above. For each spectrum a total of 120 scans (900–4000 cm^−1^) were averaged. Background of the internal reflection element was collected and subtracted to the samples. The determination of protein secondary structures was performed by deconvolution of the curve-fitting of the amide I band with Lorentzian functions.

### Bias-exchange metadynamics simulations in water

In order to properly explore the energy landscape of the WT and mutant peptides in water, we performed bias-exchange metadynamics simulations (BE-META). Metadynamics is an enhanced sampling method, in which the system is discouraged from revisiting previously sampled configurations by the application of a time-dependent external potential^34^. The bias potential acts on the space of appropriately chosen degrees of freedom called collective variables (CVs). In bias-exchange metadynamics several replicas are run in parallel, with the bias being applied to different CVs in each replica, which makes the calculations more efficient^29^. Exchanges between replicas are attempted from time to time and accepted or rejected with a probability given by a Metropolis criterion.

Before performing the BE-META simulations in water, an extended conformation of the WT and mutant fusion peptides was built using PyMOL^35^. Each peptide was then solvated in a dodecahedral water box with explicit water molecules, considering a minimum distance of 1 nm between the peptide and the box walls. These systems were simulated for 5 ns without applying any bias, with GROMACS 4.0.4^36^,^37^, using the GROMOS 54A7 FF^38^ and SPC parameters^39^ to describe the peptide and water molecules, respectively. The simulations were performed using periodic boundary conditions at 300 K and 1 atm, with an integration time step of 0.002 ps. The temperature and pressure were kept constant with V-rescale^40^ and Berendsen^41^ coupling baths, respectively, with separate temperature coupling for the peptide and solvent. The pressure coupling constant was set to 0.5 ps and the temperature coupling constant was set to 0.1 ps. The twin-range cut-off method^42^ was applied to non-bonded interactions, with short- and long-range cut-offs of 9 Å and 14 Å, respectively. A reaction-field correction^43^ was applied to long-range electrostatic interactions, considering a dielectric constant of 54^44^. The neighbour lists were updated every 5 steps. All bonds were constrained to their equilibrium lengths with the LINCS algorithm^45^, except for water molecules, which were kept rigid with the SETTLE algorithm^46^.

The final structures of the unbiased simulations were used to initialize the BE-META simulations, which were performed with the PLUMED plugin (version 1.3)^47^ for GROMACS 4.0.4^36^,^37^, using the same setup that was described for the unbiased simulations. Eight replicas were used and each replica was biased by a different CV, namely, radius of gyration, c-alpha mean square deviation (Msd) from the NMR structure obtained in DPC micelles^13^, number of intra-protein hydrogen bonds (Hbonds), number of hydrophobic contacts (hydrophobic contacts), dihedral distance from the structure obtained in DPC micelles^13^ (Alphabeta), number of 6-residue segments that resemble an ideal alpha helix (Alpharmsd), number of pairs of 3-residue segments which are similar to the ideal antiparallel beta-sheet (Antibetarmsd), and dihedral correlation (Dihedral correlation) (the definitions of these CVs can be found in ref. 47). In order to avoid systematic errors at the boundaries, lower and upper limits were defined, beyond which the bias potential was set to 0^48^. The Gaussian width and the boundaries that were defined for each CV can be found in Table 1. Gaussian potentials with a height of 0.1 kJ/mol were added at every 10 ps and exchange attempts between replicas were made with the same time interval. A simulation length of 600 ns was used for each replica.

### Bias-exchange simulations in a DMPC membrane

In a previous work, self-assembly simulations were used to predict the most stable orientation of the influenza FP in an explicit DMPC membrane^2^. In the majority of the replicates in which the membrane spontaneously assembled, the peptide adopted a membrane-spanning configuration, which strongly perturbed the membrane and is, thus, thought to play an important role during fusion. Therefore, we used one of the membrane-spanning configurations (replicate 1) from ref. 2 as the starting point of the bias-exchange simulations. The mutants were created from the final conformation of the WT, using a slow growth method, which enabled us to introduce the mutations smoothly. After introducing the mutations, each mutant peptide was simulated for 400 ns with GROMACS 4.0.4^36^,^37^, using the GROMOS 54A7 parameters^38^ for the peptide and DMPC molecules, and the SPC model for water^39^. The simulation protocol was identical to the one described above for water simulations. However, the reference temperature in this case was 310 K, and a semi-isotropic coupling scheme was used for the pressure with a coupling constant of 1 ps.

The final coordinates from the unbiased simulations were used to initiate the BE-META simulations, maintaining the simulation conditions that were used for the unbiased simulations. The BE-META protocol was similar to the one described above for BE-META simulations in water, although 9 CVs were used in this case. The dihedral correlation was replaced by the number of contacts between helices (Helix contacts) and a CV which measures the minimum distance between the headgroups and the C-terminal group (Mindist) was added. The Gaussian width and the boundaries that were defined for each CV in the membrane simulations can be found in Table 2. A simulation length of 700 ns was used for each replica. In order to increase the sampling efficiency in the highly viscous membrane environment, all the heavy atoms masses were scaled by a factor of 1/10. Although a kinetic model was built in the current work, this model was constructed by using the free energies estimated from the BE simulations and, thus, is not affected by the scaling of the masses.

### Analysis of Bias-exchange simulations

The METAGUI tool^49^ was used to analyse the BE-META simulations. Before feeding the trajectories to METAGUI, we removed periodicity effects and fitted the trajectory to a reference structure using the GROMACS tool trjconv.[8] Then, we used METAGUI to partition the conformations sampled in the BE-META simulations into 100 microstates (i.e. structures with similar values of the collective variables), using the k-means algorithm. The k-means algorithm, implemented in METAGUI, uses the Euclidian distance in CV space. The units of the different variables are equalized by dividing each CV by the grid spacing defined by the user (the values that were used for each CV can be found in Tables S1 and S2 in supplementary information).

The free energy of each microstate was computed by a weighted-histogram method. These microstates where then assigned to kinetic basins using the procedure described in Marinelli *et al*.^50^. We note that the kinetic model is not based on the transition probabilities observed in the simulations, since these trajectories are biased. The kinetic model is constructed by using the free energies estimated from the BE simulations

## Results

**Figure 1:**
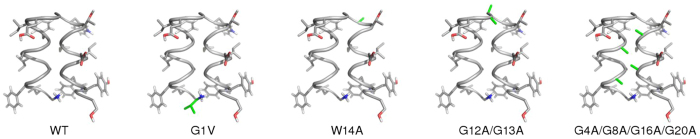
Location of the mutations on the structure of the influenza fusion peptide. The structure of the WT peptide in detergent micelles [13] is displayed in the left panel. The other panels show the location of the mutations analysed in this work, which are highlighted in green. The peptide backbone is shown with a grey cartoon representation and the residue side chains are displayed as sticks with carbon, oxygen, nitrogen and hydrogen atoms coloured in grey, red, blue and white, respectively.

The main goal of this work was characterizing the structural properties of the influenza FP and its effect on model membranes, using a combination of theoretical and experimental methodologies. Moreover, since mutations in several amino acid residues have been shown to impair or inactivate its function, we also aimed to study the effect of mutations. We selected two point mutations (G1V and W14A) that have been shown to significantly reduce the HA activity^18^,^21^. In order to determine the effect of mutations in the turn region, we also analysed the mutant G12A/G13A. Finally, we constructed a mutant, in which glycine residues 4, 8, 16 and 20 (which form the glycine zipper motif that seems to be crucial to stabilize the closed helical-hairpin structure^13^) were replaced by alanine residues (mutant G4A/G8A/G16A/G20A). The location of the mutations that were analysed in this work is shown in Fig. 1.

### Structural properties of the wild-type and mutant fusion peptides in water analysed by BE-META simulations

In the prefusion structure of hemagglutinin, the FP is enclosed inside a hydrophobic pocket of the protein. Upon exposure to the low pH of late endosomes, the FP is extruded from this pocket and becomes exposed to water, before it inserts into the host membrane. In order to analyse the conformational properties of the WT and mutant peptides at this stage of the fusion process, we first performed BE-META simulations in water. This methodology was chosen since it is considerably more efficient than standard MD and can provide a detailed description of the free energy landscape of small peptides^30^.

**Figure 2:**
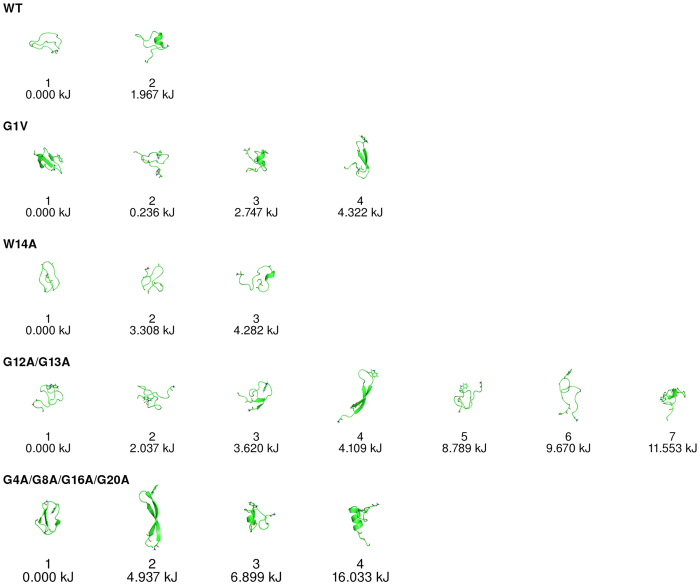
Kinetic basins computed from the BE-META simulations of the WT peptide and its mutants in water. In order to compute the kinetic basins the conformations sampled in the BE-META simulations were projected onto the multidimensional space defined by a set of CVs (Radius of gyration, Alphabeta, Alpharmsd, Betarmsd, Hbond, Number of Hydrophobic contacts and Dihedral correlation) and grouped into microstates using the k-means algorithm implemented in METAGUI^49^. The relative free energy of each cluster was estimated by a weighted histogram procedure and each cluster was then assigned to a kinetic basin, as described in Marinelli *et al*.^50^.

The 1D free energy profiles of all the CVs that were used in this study are quite flat both for the WT and mutants (see Figs S1–S5 in supplementary information), indicating that the peptides adopt several different conformations. In order to gain further insight into the structural properties of the WT and mutant peptides, we built a multidimensional free energy landscape of each peptide using the method described by Marinelli *et al*.^50^. This method generates a series of microstates which are then grouped into kinetic basins (Fig. 2).

**Figure 3:**
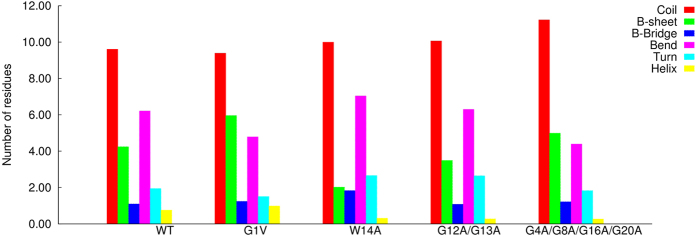
Secondary structure content of the WT and mutant fusion peptides in BE-META simulations performed in water. The secondary structure of each microstate was computed with DSSP and then a weighted average over all the microstate was calculated (the weight of each microstate was obtained from its free energy). We note that the category named “helix” encompasses all types of helical structures.

The results obtained show that the WT peptide populated two distinct basins: one which encompasses completely random coil conformations (basin 1) and another one in which a short helical segment is present (basin 2). The mutant G1V adopted two different types of β-sheet conformations (basins 1 and 4), as well as completely unstructured states (basin 2) and conformations containing a small helical segment (basin 3). The mutant W14A populated mainly random coil basins and the same was observed for the mutant G12A/G13A, although in this case β-sheet conformations were also sampled. The mutant G4A/G8A/G16A/G20A adopted both random coil and β-sheet conformations. A basin containing structures characterized by a helical segment was also present in this mutant, although its free energy was high. The analyses of the average secondary structure content of each peptide revealed that all the peptides were predominantly unstructured in water simulations (Fig. 3).

**Figure 4:**
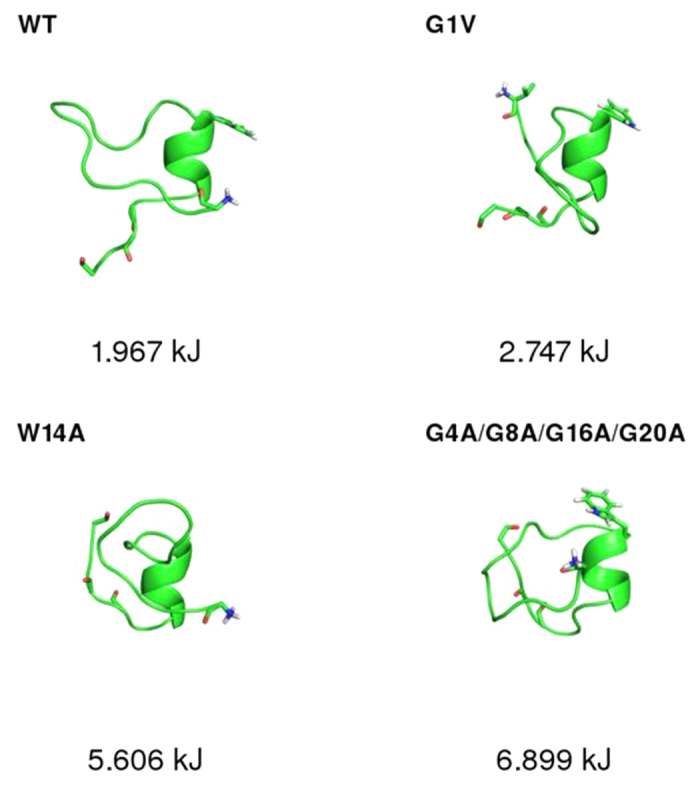
Conformations in which the C-terminal helix observed in the structure obtained in DPC micelles^13^ are partially formed. For each peptide, the lowest free energy microstate containing the helical fragment partially assembled is shown. This type of conformation was not observed in the mutant G12A/G13A.

A key question that can be addressed by analysing the free energy landscapes of the WT and of the mutants is whether the FP can adopt conformations that resemble the helical structure obtained in DPC micelles^13^ before it enters the host membrane. We observed that, although this particular structure was not significantly populated in any of the peptides, the WT peptide adopted a conformation in which the C-terminal helix was partially formed (Fig. 4). This microstate had a relative free energy of ~2 kJ, which means that it is significantly populated at room temperature. The G1V, W14A and G4A/G8A/G16A/G20A mutants also populated a similar state, although in this case the relative free energy was larger than in the case of the WT. The mutant G12A/G13A did not acquire this type of conformation, which indicates that that the glycine residues in the kink are critical for the formation of this helical segment.

### Structural properties of the wild-type and mutant fusion peptides in a lipid membrane

In order to analyse the free energy landscape of the WT and mutant peptides in a membrane environment, we performed BE-META simulations in a DMPC bilayer. The 1D free energy profiles obtained for the WT FP are characterized by clear free energy minima (Fig. S6 in supplementary information), meaning that in the membrane the peptide adopts well-defined and stable conformation, which contrasts with the results obtained in water.

**Figure 5:**
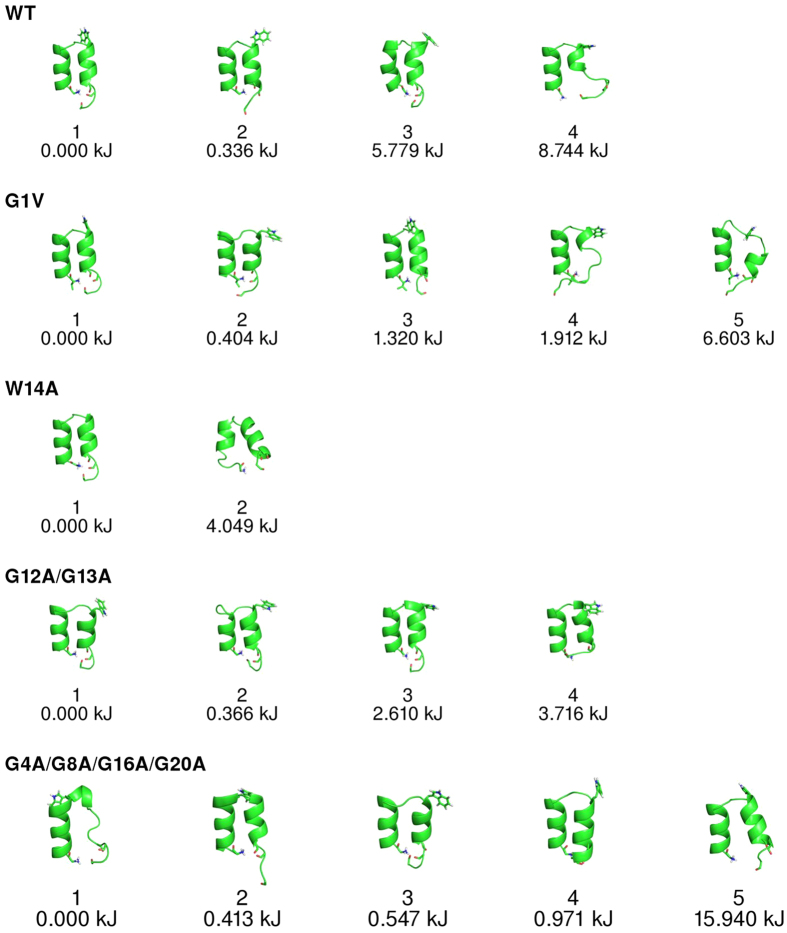
Kinetic basins computed from the BE-META simulations of the WT peptide and its mutants in a DMPC membrane. The basins were computed as described in Fig. 2 and the CVs used in the analysis were: Alphabeta, Alpharmsd, Betarmsd, Hbonds and Number of hydrophobic contacts.

Similarly to what was done for the simulations in water, we analysed the multidimensional free energy landscape of the peptide in the membrane. As can be seen in Fig. 5 (first row), the WT peptide populates 4 basins, with the lowest energy states (basins 1 and 2) being very similar to the helical-hairpin NMR structure obtained in detergent micelles (see Fig. 1)^13^. The only feature that changed among these two basins was the interaction between the terminals. Basins 3 and 4 correspond to high free energy states, which indicates that the helical-hairpin structure is very stable.

We then analysed the free energy landscape of the mutants and observed that the mutant G1V adopted mainly helical-hairpin conformations similar to the WT (basins 1 and 3) (Fig. 5, second row). However, in this mutant we also observed low free energy states in which the C-terminal helix was almost completely absent (basin 4), indicating that the G1V mutation disturbs the peptide structure. The lowest free energy conformation of the mutant W14A was similar to that observed for the WT (basin 1), although this mutant also populated more open conformations (basin 2). As observed for the WT, the kinetic basins obtained for the G12A/G13A encompass mostly helical-turn-helical conformations. However, the turn region, where the mutations are located, was more unstable than in the case of the WT. The arrangement of this region varied among basins, becoming quite distorted in some cases. These findings indicate that the residues G12 and G13 are important to maintain the stability of the turn region.

The free energy landscape of the G4A/G8A/G16A/G20A mutant was the one that differed more significantly from that of the WT. This mutant is considerable more unstable than the WT, populating distinct kinetic basins. In the lowest free energy state, the C-terminal helix is completely destroyed, whereas in other low energy states, the peptide adopted helix-turn-helix structures, in which the helices tended to be considerably more distant from each other than in the case of the WT and have a lower contact area. These results support the hypothesis that the glycine zipper formed by glycine residues 4, 8, 16 and 20 is essential to stabilize the fusion peptide structure^13^.

The effect of the mutants on the stability of the helical-hairpin structure can be captured by comparing the WT and mutant free energy profiles of the CV ALPHARMSD (Fig. S11 available in the supporting information). This CV measures the peptides’ helical content and, thus, can distinguish folded from partially folded or unfolded structures. As can be seen in this figure, the WT peptide has a “downhill profile”, displaying only one minimum, which corresponds to the helical-hairpin structure. The mutants’ profiles, on the other hand, have more than one minimum. In the case of the G4A/G8A/G16A/G20A, the landscape is quite flat and has two minima with very similar energy values.

**Figure 6:**
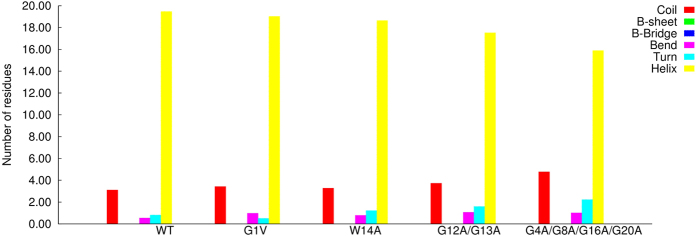
Secondary structure content of the WT and mutant fusion peptides in BE-META simulations performed in a DMPC membrane. The calculations were performed as described in Fig. 3.

The average secondary structure content of each peptide in the membrane environment was calculated (as described for the simulations performed in water) and this analysis showed that all the peptides were mainly helical (the term helical is used to refer to all types of helical structures, i.e. alpha-helix, 3_10_ helix, π helix) (Fig. 6), although the mutant G4A/G8A/G16A/G20A displayed a smaller helical content than the WT.

### Effect of the WT and mutant peptides on the membrane properties

**Figure 7:**
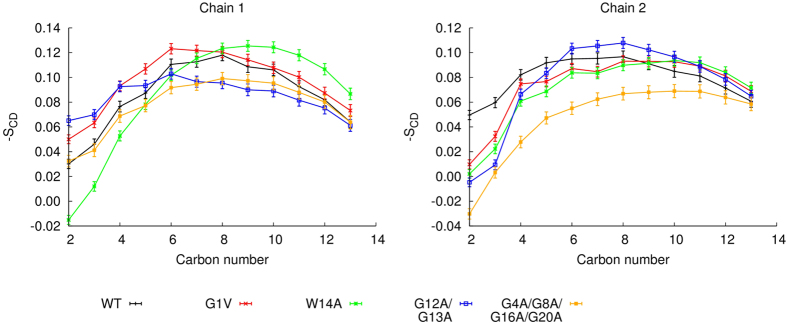
Order parameters of lipids that are in contact with the peptides computed from the BE-META simulations in a DMPC membrane. The order parameters for the sn-1 (left panel) and sn-2 (right panel) acyl tails of DMPC are shown. The order parameters were calculated with the g-order tool available in the GROMACS 4.0.4 package^37^. Only the lipids which were within a distance of 0.5 nm from the peptides were included in the calculation. The plots shown correspond to a weighted average over all the microstates (the weight of each microstate was obtained from its relative free energy). The errors were obtained by calculating the standard error for each cluster and using the error propagation method to estimate the global errors.

One of the objectives of this work was to compare the effect of the WT and mutant peptides on the membrane properties. Previous MD simulation studies^4^,^25^,^51^, including a recent study performed by us^2^, have found that the fusion peptide causes a decrease in the order of the surrounding lipids. Therefore, we compared the effect of the WT and mutant peptides in the order parameters of adjacent lipids. The calculated values correspond to a weighted average over all the microstates obtained in BE-META simulations, in which the weight of each microstate is obtained from its free energy. Our results indicate that the G1V, W14A and G12A/G13A mutants had a similar effect to the WT peptide on the order of the surrounding lipids, whereas the G4A/G8A/G16A/G20A mutant had a stronger influence on the order parameters of the acyl chain 2 (Fig. 7)

**Figure 8:**
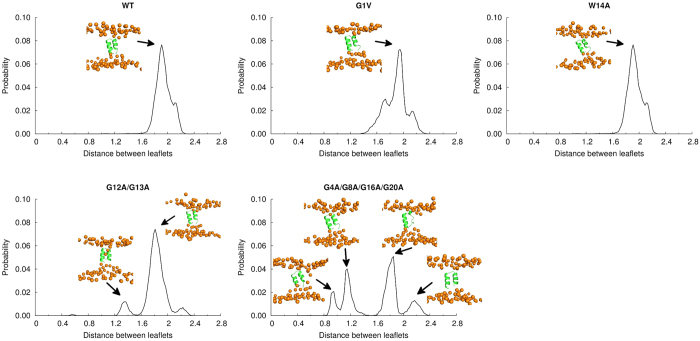
Distribution of the minimum distance between bilayer leaflets computed from the BE-META simulations in a DMPC membrane. The distributions correspond to a weighted average over all the microstates (the weight of each microstate was obtained from its free energy).

It has also been previously observed that the FP attracts the lipid headgroups, which induces a decrease in the distance between membrane leaflets^2^,^52^. In order to compare the effect of the WT and mutant peptides, we plotted the distribution of the minimum distance between phosphates in the upper and lower leaflets (Fig. 8). In the presence of the WT peptide, the distribution of the minimum distance between leaflets had a sharp peak centred at ~1.9 nm and a similar profile was observed in the case of the G1V and W14A mutants. This peak was also observed in the simulations performed with the mutant G12A/G13A, although in this case another peak of smaller size was observed at shorter distances. This indicates that the mutant G12A/G13A has a stronger effect on the membrane and can pull the headgroups of the two leaflets closer to each other, as can be observed in the snapshot configuration shown on the inset of the plot. In the plot corresponding to the G4A/G8A/G16A/G20A mutant 4 peaks can be found. The largest peak is located at around ~1.9 nm as is observed in the WT case. There is a small peak on the right that corresponds to conformations in which the peptide has a small effect on the lipid headgroups. Additionally, there are two other peaks cantered at distances below 1.2 nm. The left-most peak corresponds to very short distances between leaflets, as can be seen in the inset, indicating that this mutant can have a very pronounced effect on the membrane, inducing, in some cases, contact between the head groups of lipids from opposing leaflets. The large number of peaks found for this mutant can be attributed to the fact that it has a less well-defined free energy landscape, including a few structurally different kinetic basins.

**Figure 9:**
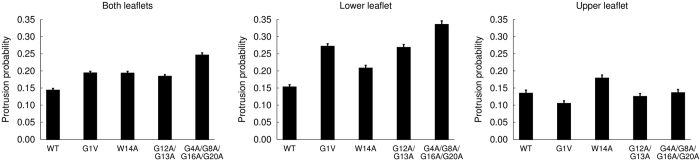
Probability of lipid tail protrusion of lipids that are in contact with the peptides. Lipid protrusion was assumed to occur when any carbon in the lipid tail protruded more than 0.1 nm beyond the phosphate group. Only the lipids which were within a distance of 0.5 nm from the peptides were included in the calculation. The plots shown correspond to a weighted average over all the microstates (the weight of each microstate was obtained from its free energy). The errors were calculated as described in Fig. 7.

In previous MD simulations^3^,^4^,^52^, the influenza FP has been found to promote lipid tail protrusion of nearby lipids due to its ability to attract the lipid headgroups, Protrusion occurs when a carbon from one of the lipid tails extends beyond the corresponding phosphate group. This effect is proposed to play an important role in the fusion process^3^. We compared the ability of the WT and mutant fusion peptides to induce lipid tail protrusion and observed that the probability of observing this event in the presence of the mutants is higher than in the presence of the WT (Fig. 9). When the protrusion probability is analysed separately for the upper and lower leaflets, it becomes apparent that the mutants have a larger effect on the lower leaflet relative to the WT, whereas the probabilities are similar for the upper leaflet, except for the mutant W14A.

### Secondary structure of the FP WT and mutant peptides analysed by FTIR

In order to complement the computational analysis described above, we used a series of experimental techniques to study the structure and activity of the WT and mutant peptides.

**Figure 10:**
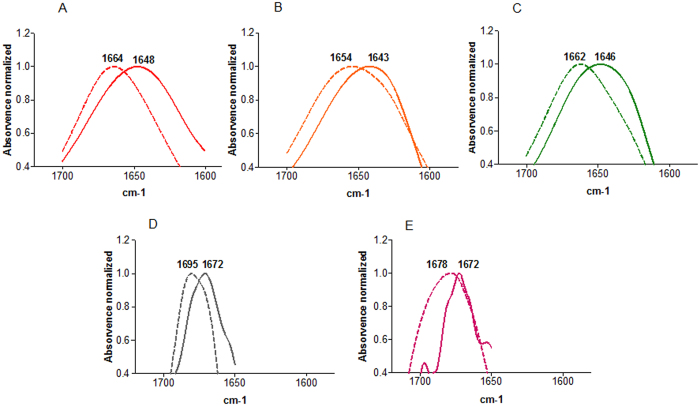
ATR-FTIR amide I band for the WT FP (**A**) and mutant peptides (**B**), G1V; (**C**) W14A; (**D**) G12A/G13A; (**E**) G4A/G8A/G16A/G20A) in aqueous solution (solid line) or in presence of POPC LUV (dashed line). All the spectra were normalized.

We started by analysing the peptides’ secondary structure by ATR-FTIR spectroscopy. The spectra of the WT and mutant peptides in the absence and presence of lipidic membranes were collected at pH 5.0 (Fig. 10). For the WT, G1V and W14A the wavenumber ranges of the amide I absorption bands indicate that these peptides are mainly random coil in aqueous solution. On the other hand the results obtained for the G4A/G8A/G16A/G20A and G12A/G13A mutants show that they tend to adopt β-sheet conformations.

In the presence of POPC membranes, the ATR-FTIR spectra obtained show that the WT, G1V and W14A peptides have a different structure when compared to the one adopted in aqueous solution, being mainly in a helical conformation (Fig. 10). The spectra obtained for the G4A/G8A/G16A/G20A and G12A/G13A mutants are typical of antiparallel β-sheet conformation or aggregated strands.

### Partition coefficients of the FP WT and mutant peptides measured by intrinsic fluorescence spectroscopy

Since all studied peptides are intrinsically fluorescent due to the presence of tryptophan residues, fluorescence emission spectroscopy was used to study the interaction of the peptides with POPC/POPE LUVs, at endosome mimetic pH (5.0). The fluorescence quantum yield is dependent on the polarity of the microenvironment of the tryptophan residues, which is affected upon insertion of the peptides in membranes. The partition coefficients (*K*_*p*_) between the aqueous and lipid phases were determined to quantify the extent of the peptides incorporation in LUV bilayers and are shown in Table 3. The results obtained show that the mutants W14A, G12A/G13A and G4A/G8A/G16A/G20A have a decreased affinity for membranes when compared to the WT FP (Table 3).The partition coefficient of the G1V mutant could not be determined.

Using the partition coefficient, we calculated the concentration of each peptide in the lipid vesicles as described in ref. 53 (Table 3). The calculated values show that the concentration of the mutants G12A/G13A and G4A/G8A/G16A/G20A in the lipid is around half the concentration of the WT. On the other hand, the concentration of the W14A mutant was higher than the one obtained for the WT. However, we note that the total peptide concentration used in the W14A assays was higher than the one that was used for the other peptides, which accounts for the observed increase in the concentration of peptide in the lipid.

### Fusogenic activities of the FP WT and mutant peptides analysed by a FRET-based assay

In order to study the peptides’ fusogenic activity, a FRET-based assay was performed using POPC/POPE LUV at pH 5.0 and the percentage of fusion efficiency was obtained. The results (Table 4) show that the highest membrane fusion efficiency was observed for the WT peptide. All the mutant peptides showed lower fusion activity when compared to the WT and the effect was particularly pronounced in the case of the mutant G4A/G8A/G16A/G20A. Globally, the ability of the peptides to induce lipid mixing decreased in the order WT > G1V > W14A > G12A/G13A > G4A/G8A/G16A/G20A.

## Discussion

The main goal of this study was to shed light into the structural and fusogenic properties of the influenza fusion peptide by using a combination of experimental and simulation methodologies. In addition to the WT peptide, we also analysed four mutant fusion peptides (G1V, W14A, G12A/G13A and G4A/G8A/G16A/G20A), in order to determine how these mutations affect the FP structure and activity.

Since there is a strong correlation between structure and function, characterizing the conformational properties of the FP is crucial to understand its role in the fusion process. To address this question, we used a BE-META simulation approach, which allowed us to circumvent the limitations inherent to standard MD and characterize the free energy landscape of the WT and mutant peptides, both in water and in a membrane bilayer, which, to the best of our knowledge, had never been done before.

We started by performing BE-META simulations of the peptides in water, given that the FP is exposed to this media before it inserts into the host membrane. According to the BE-META simulations, all the peptides were mainly unstructured in aqueous solution. These results are in agreement with our ATR-FTIR studies for the WT and the mutants G1V and W14A. In the case of the mutants G12A/G13A and G4A/G8A/G16A/G20A there is some apparent discrepancy between the experimental and theoretical results, since their ATR-FITR spectra are typical of β-sheet conformations. This discrepancy is most likely due to the β-sheet aggregates which tend to form in the experimental procedures, but are obviously not observed in the simulations, where only one peptide copy was simulated.

The BE-META simulations in water revealed that the WT fusion peptide samples partially helical conformations, which share some common features with the conformation observed in DPC micelles^13^. This is a very interesting finding, since it suggests that the FP explores structures that are partially akin to the ones they take in the membrane, which may facilitate the fusion process. Moreover, our results indicate that all the mutations studied destabilized these “partially pre-assembled” conformations. In the G12A/G13A mutant, the helical segment could not form at all, indicating that these residues are crucial for the formation of this helix in water.

One of the most important goals of this study was to analyse the free energy landscape of the fusion peptide in a membrane bilayer, since it is known that this peptide inserts into the host cell membrane during the fusion process. Although the NMR structure of this peptide in DPC micelles is available, it is still not clear if this structure corresponds to the most stable conformation in a membrane bilayer, which is a different environment. MD simulations have been previously used to address this question, including a previous work by our group, where we performed simulations in which a membrane bilayer spontaneously assembled around the peptide. In that study we used the NMR structure determined by Lorieau *et al*. in DPC micelles^13^ and observed that the peptide maintained its structural arrangement during the simulations. However, since the membrane is a very viscous environment, we could not determine whether this structure corresponds to the most stable conformation in a membrane bilayer or if it was only a metastable state. In the current work, to answer this question unambiguously, we performed bias-exchange metadynamics simulations of the influenza FP in a membrane bilayer. This method is more powerful than standard MD simulations, since it allows us to extensively explore the free energy landscape of the peptide and find which are the most populated conformations and their relative free energies.

The BE-META simulations performed in a model membrane showed that the most stable states of the WT FP in the membrane correspond to helical-turn-helical structures, very similar to the NMR structure obtained in DPC micelles^13^, which is consistent with the results obtained in our previous work where this structure was found to be stable^2^. Importantly, since metadynamics provides an estimation of the relative free energies of different conformational states, we were able to show for the first time that the helical-hairpin conformation is much more stable than the other states sampled by this peptide.

All the mutations studied had an effect on the energy landscape of the FP in the membrane, increasing the probability of observing conformations distinct from the NMR structure, which indicates that the residues that were mutated are crucial to maintain the FP stability. Of all the mutations analysed, the G4A/G8A/G16A/G20A is the one that has a stronger effect on the peptide structure, inducing very distorted conformations. This supports the hypothesis that the glycine residues 4, 8, 16 and 20 are important to stabilize the FP structure, as suggested by Lorieau *et al*.^13^ Our BE-META simulations also support the notion that glycine residues 12 and 13 are important to maintain the stability of the kink region, since this region is destabilized in the G12A/G13A mutant. The mutants G1V and W14A have less pronounced effects on the peptide structure, although the G1V mutation results in the appearance of distorted structures with low free energy values.

The simulations predicted that all the peptides adopt mainly helical conformations. These results are consistent with the ones obtained experimentally, using ATR-FTIR, in the presence of POPC membranes for the WT, G1V and W14A peptides. However, the ATR-FTIR spectra of the mutants G4A/G8A/G16A/G20A and G12A/G13A were typical of structures with high β-sheet content. The apparent discrepancy between these results is likely due to the fact that these peptides tend to aggregate, which can affect their secondary structure. Thus, our hypothesis is that the G12A/G13A and G4A/G8A/G16A/G20A mutants are helical when they are in a monomeric state in the membrane. According to our simulations, their structure is not as stable as that of the WT peptide, so they may have a high propensity to form beta-sheet aggregates under the experimental conditions of the ATR-FTIR assays.

The effect of the WT and mutant peptides on the membrane was compared by analysing how different membrane properties, such as the distance between leaflets, the lipid order parameters and the occurrence of lipid tail protrusion were affected by the peptides’ presence. The results of these analyses suggest that all the mutants increase the probability of lipid tail protrusion relative to the WT peptide. The G4A/G8A/G16A/G20A mutant also slightly increases the disorder of nearby lipids and the probability of contact between opposing leaflets. Overall, this peptide is the one which has a stronger effect on the membrane. This is consistent with the fact that this mutant adopts more distorted and open conformations than the WT peptide in the membrane.

In order to complement the results obtained in the BE-META simulations, we used FRET-based assays to measure the peptides’ ability to induce lipid-mixing, which showed that the mutant peptides had lower activity than the WT fusion peptide and the ability to induce lipid mixing decreased in the order WT > G1V > W14A > G12A/G13A > G4A/G8A/G16A/G20A. Overall there appears to be correlation between the effect of the mutations studied on the FP energy landscape and their effect on the peptide’s activity. This clear correlation indicates that the fusogenic activity of influenza FP requires a stable helical-hairpin structure.

Given that, according to our simulations, the mutant peptides have a similar or larger effect on the membrane properties (lipid order, distance between lipids and lipid tail protrusion), the fact that they are less efficient in promoting lipid-mixing than the WT must be attributed to other factors.

Fluorescence-based assays showed that the mutants G12A/G13A and G4A/G8A/G16A/G20A, which are the less active peptides, have a considerably lower affinity for the membrane. According to our calculations, the concentration of these peptides in the membrane is around half that of the WT FP. This indicates that loss of efficiency observed for these peptides is, at least partially, due to the lower number of peptides present in the membrane. This is consistent with previous experimental evidences showing that the fusion process requires the concerted action of several FP monomers^54^,^55^. These results are also in line with the observations of coarse-grained MD simulations^26^,^56^. Thus, one important point that needs to be further addressed in the future is the analysis of the interaction between FP monomers and its effect on the membrane properties.

The present work also revealed other factors that may contribute to the lower activity of the mutants, namely, the lower probability of sampling partially assembled conformations in water, the destabilization of the helical-hairpin structure in the membrane and the formation of beta-sheet aggregates (in the case of the G12A/G13A and G4A/G8A/G16A/G20A mutants).

These results provide important insights into the structural and fusogenic properties of the influenza peptide and generate new hypothesis which will foster future studies.

## Additional Information

**How to cite this article**: Lousa, D. *et al*. Fusing simulation and experiment: The effect of mutations on the structure and activity of the influenza fusion peptide. *Sci. Rep.* **6**, 28099; doi: 10.1038/srep28099 (2016).

## Supplementary Material

Supplementary Information

## Figures and Tables

**Table 1:** Parameters used in BE-META simulations in water.

	Radius of gyration	MSD	Hbonds	Hydrophobic contacts	Alphabeta	Alpharmsd	Antibetarmsd	Dihedral correlation
Sigma	0.05	0.05	2	20	0.5	0.2	0.2	0.2
Interval	0.6–1.2	0.2–0.8	28–48	430–570	1–15	0.2–6	0.2–6	10–25

**Table 2:** Parameters used in BE-META simulations in the DMPC membrane.

	Radius of gyration	MSD	Hbonds	Hydrophobic contacts	Alphabeta	Alpharmsd	Antibetarmsd	Helix Contacts	Mindist
Sigma	0.002	0.002	0.5	10	0.05	0.2	0.2	0.15	0.025
Interval	0.65–0.75	0.02–0.1	38–50	420–530	18–21	0.2–13	0.2–6	1–5	0.1–0.5

**Table 3:** Lipid/water partition coefficients.

Peptide	K_p_	Total Peptide concentration used (mM)	Peptide concentration in the lipid (mM)
WT	10455	0.016	5.08
W14A	6265	0.029	9.03
G1V	–	0.016	–
G12AG13A	442	0.016	3.01
G4AG8AG16AG20A	333	0.016	2.65

**Table 4:** Efficiency of lipid mixing.

Peptide	*%Eff. FRET*
WT	42.8 ± 1,7
W14A	14.2 ± 3.8
G1V	22.8 ± 1,1
G12AG13A	11.3 ± 6.5
G4AG8AG16AG20A	3.2 ± 0.8
