# Supplementary material for: Fusing simulation and experiment: The effect of mutations on the structure and activity of the influenza fusion peptide
Source: Sci Rep. 2016 Jun 15;6:28099. doi: 10.1038/srep28099 (PMC4908596; doi:10.1038/srep28099)

Supplementary Information

Fusing simulation and experiment: The effect of mutations on the structure and activity of the influenza fusion peptide

Diana Lousa, Antónia R. T. Pinto, Bruno L. Victor, Alessandro Laio, Ana S. Veiga, Miguel A. R. B. Castanho* and Cláudio M. Soares*

Tables

Table S1. Parameters used for the analysis of BE-META simulations in water. The table shows the CVs that were used to partition the structures sampled in the simulations into microstates. For each CV, the boundaries and the number of grid points used for the analysis are shown.

		MSD	Hbonds	Alphabeta	Alpharmsd	Dihedral correlation
Boundaries	WT	0.2 – 0.75	28 - 48	1 - 15	0.2 - 6	10 - 25
	G1V	0.2 – 0.75	28 - 48	1 - 15	0.2 - 6	10 - 25
	W14A	0.2 – 0.75	28 - 48	1 - 15	0.2 - 6	10 - 25
	G12A/G13A	0.2 – 0.75	28 - 48	1 - 15	0.2 - 6	10 - 25
	G4A/G8A/G16A/G20A	0.2 – 0.75	28 - 48	1 - 15	0.2 - 6	10 - 25
Number of grid points		15	15	15	15	15

Table S2. Parameters used for the analysis of BE-META simulations in a DMPC membrane. The table shows the CVs that were used to partition the structures into microstates. For each CV, the value of the boundaries and the number of grid points used for the analysis are shown.

		Hydrophobic contacts	Hbonds	Alphabeta	Alpharmsd	Betarmsd
Boundaries	WT	420 – 530	38 – 50	18 - 21	8 - 13	0.2 - 6
	G1V	420 – 530	38 – 50	18 - 21	8 - 13	0.2 - 6
	W14A	350– 500	38 – 50	18 - 21	8 - 13	0.2 - 6
	G12A/G13A	420 – 530	38 – 50	18 - 21	7 - 13	0.2 - 6
	G4A/G8A/G16A/G20A	420 – 530	38 – 50	18 - 21	4 – 9.5	0.2 - 6
Number of grid points		12	12	12	12	12

Figures

Figure S1. Free energy profiles calculated from BE-META simulations of the WT peptide in water. The red and green lines correspond to the profiles obtained after 575 and 600 ns of simulation, respectively.

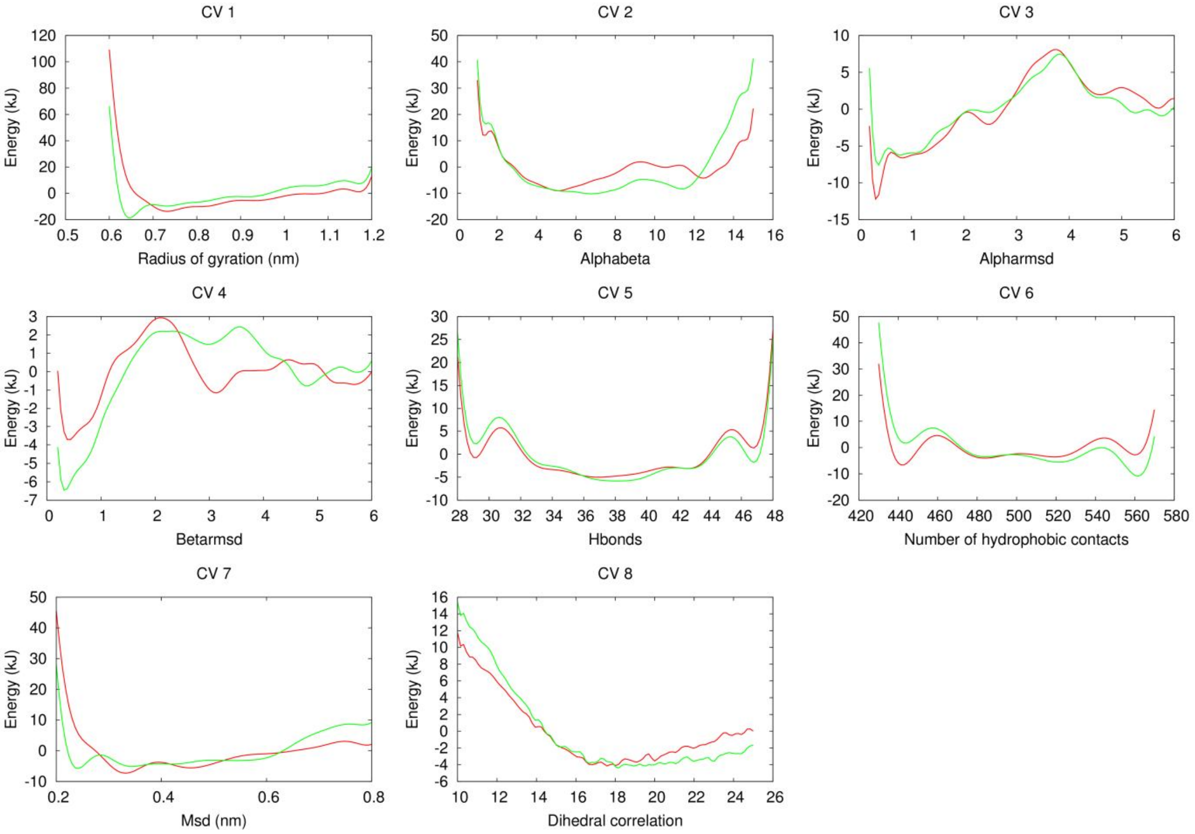

Figure S2. Free energy profiles calculated from BE-META simulations of the mutant G1V in water. The red and green lines correspond to the profiles obtained after 575 and 600 ns of simulation, respectively.

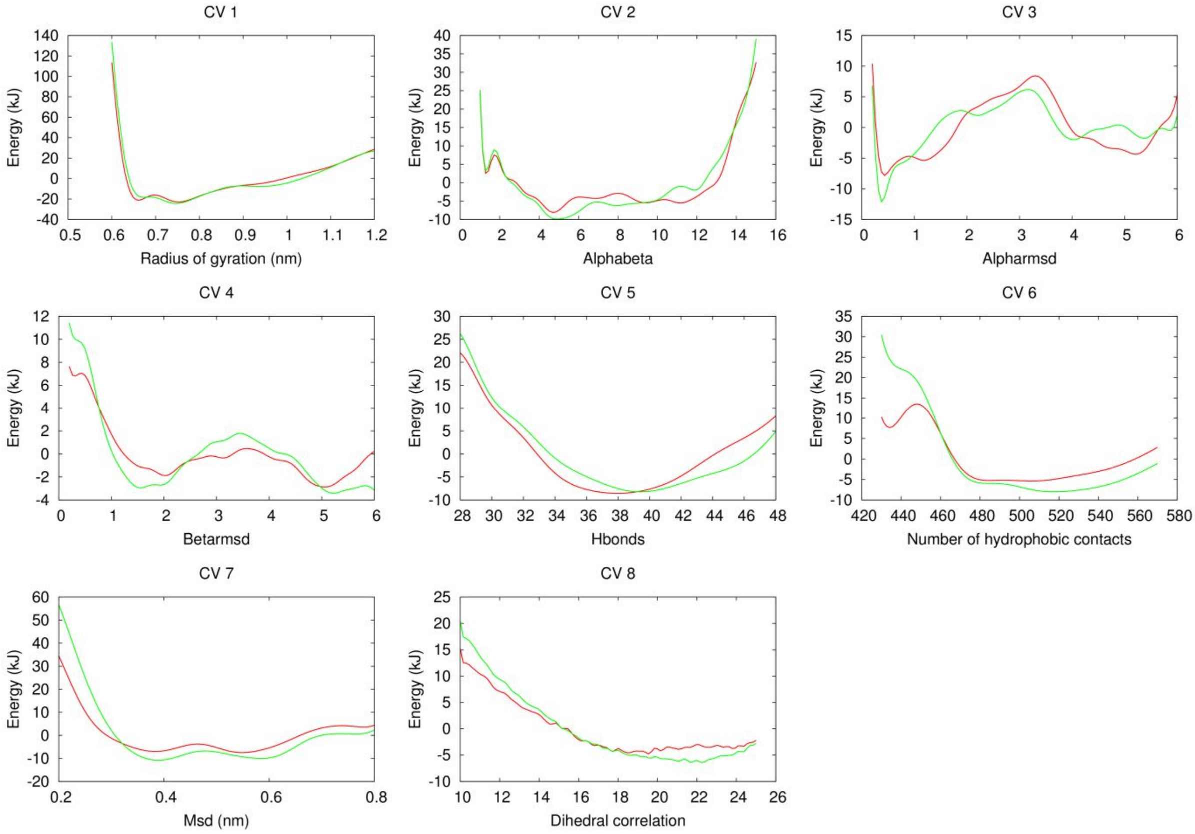

Figure S3. Free energy profiles calculated from BE-META simulations of the mutant W14A in water. The red and green lines correspond to the profiles obtained after 575 and 600 ns of simulation, respectively.

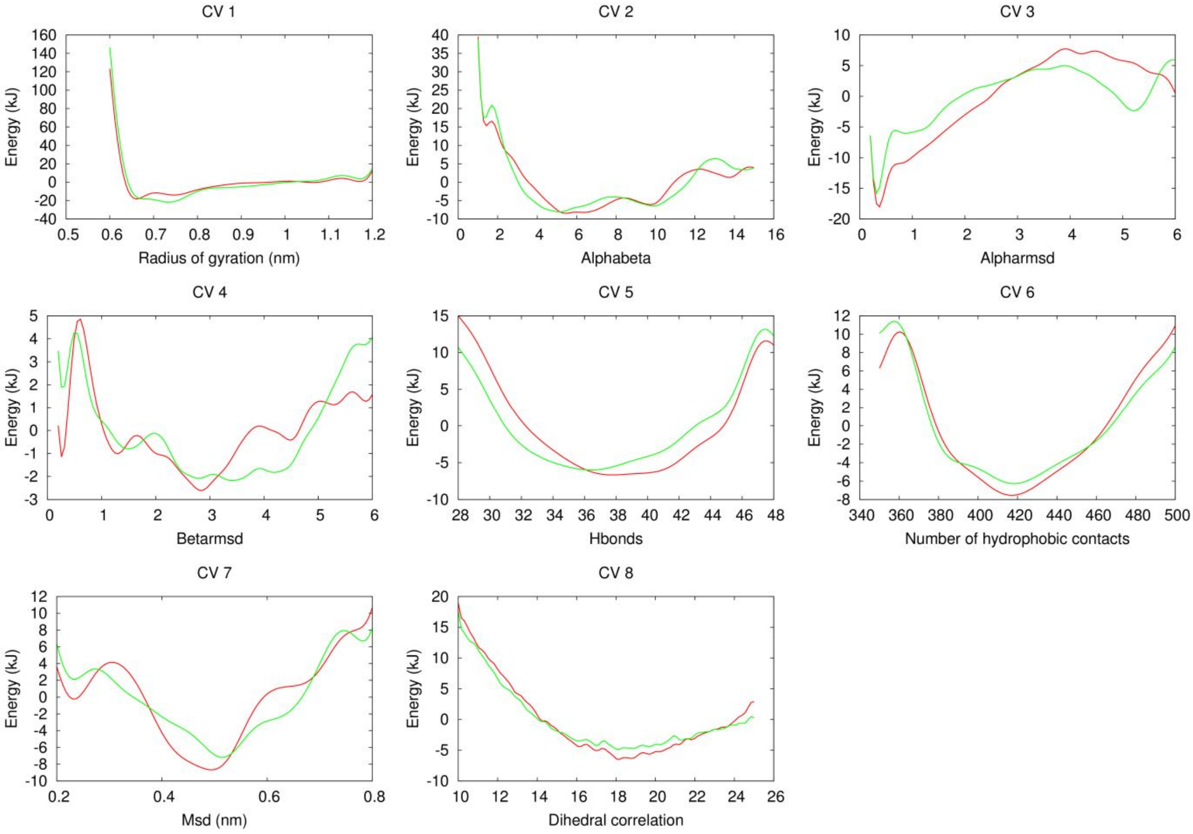

Figure S4. Free energy profiles calculated from BE-META simulations of the mutant G12A/G13A in water. The red and green lines correspond to the profiles obtained after 575 and 600 ns of simulation, respectively.

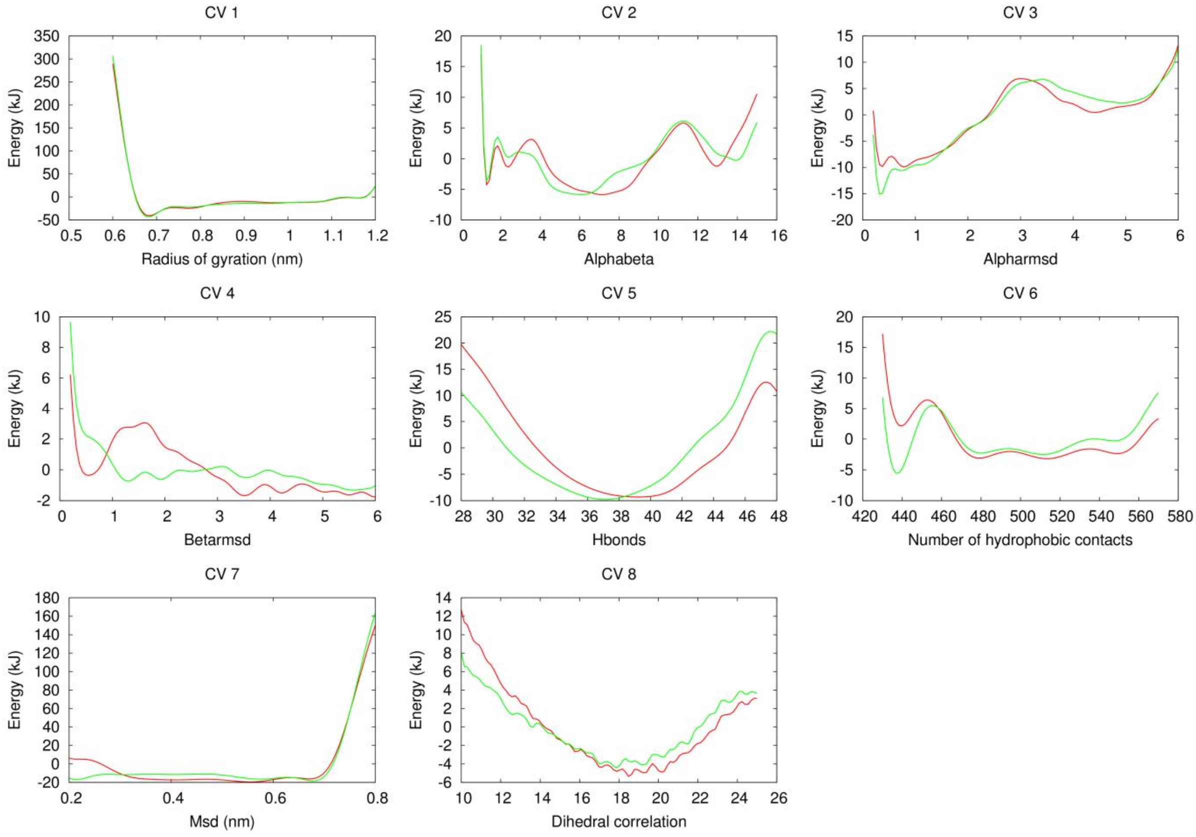

Figure S5. Free energy profiles calculated from BE-META simulations of the mutant G4A/G8A/G16A/G20A in water. The red and green lines correspond to the profiles obtained after 575 and 600 ns of simulation, respectively.

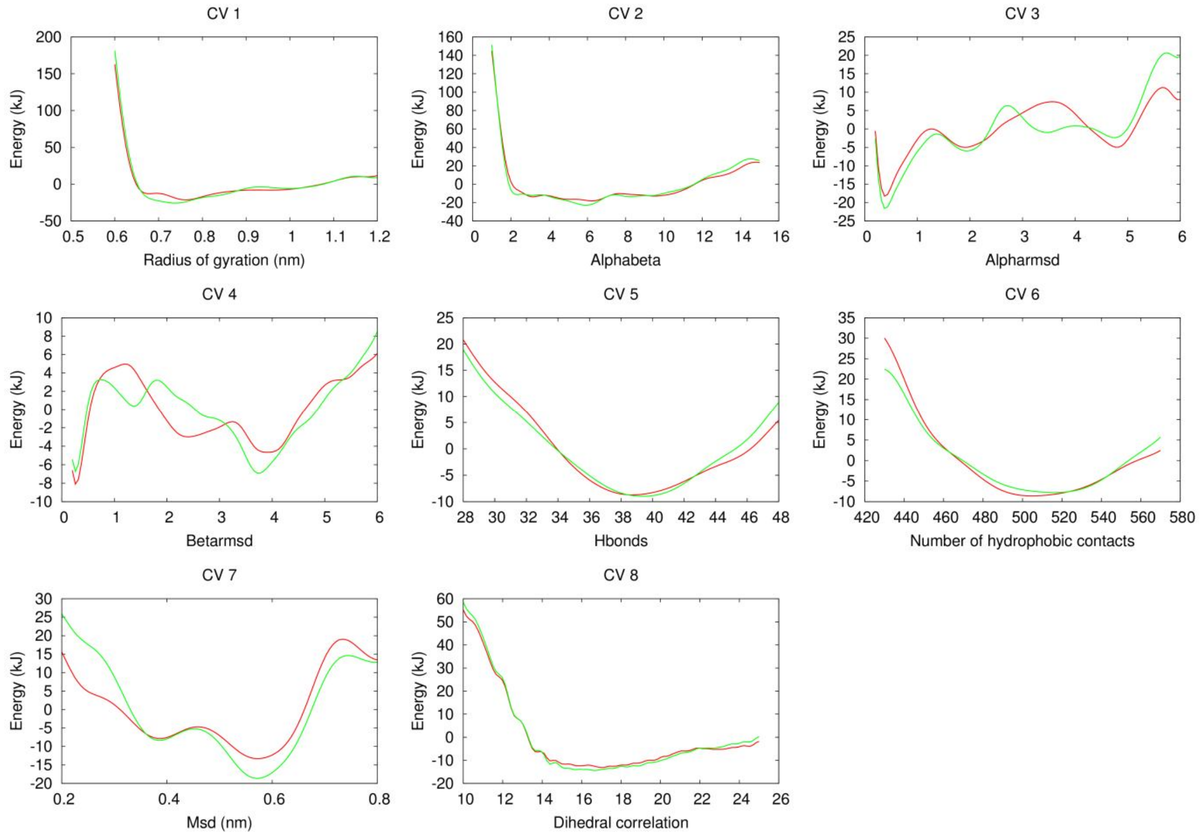

Figure S6. Free energy profiles calculated from BE-META simulations of the WT peptide in a DMPC membrane. The red and green lines correspond to the profiles obtained after 650 and 700 ns of simulation, respectively.

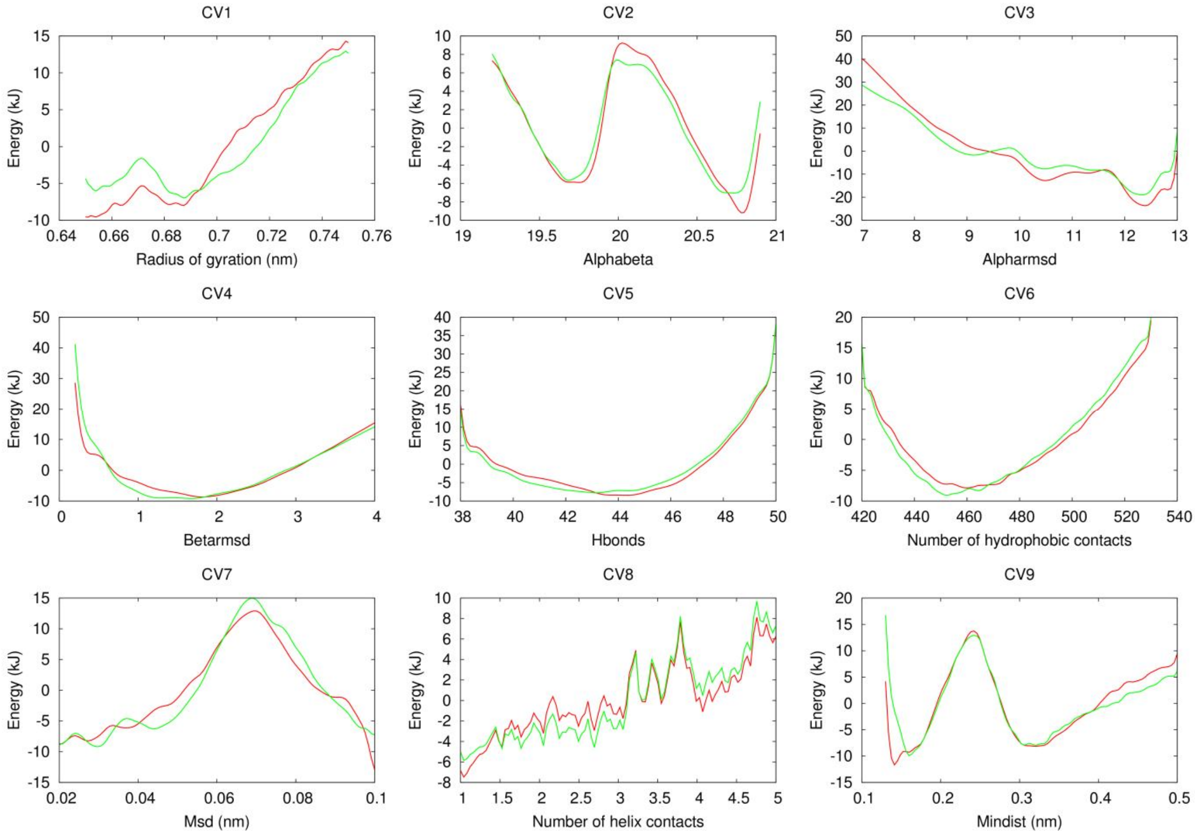

Figure S7. Free energy profiles calculated from BE-META simulations of the mutant G1V in a DMPC membrane. The red and green lines correspond to the profiles obtained after 650 and 700 ns of simulation, respectively.

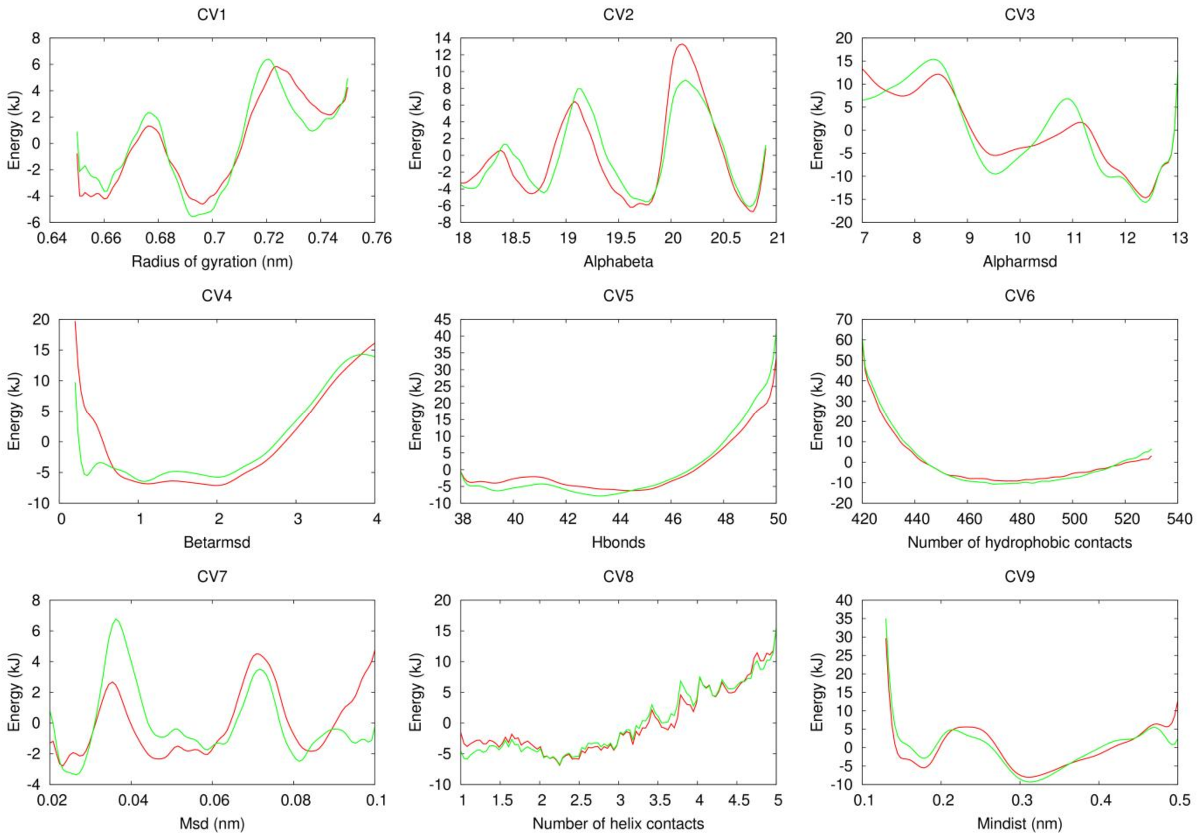

Figure S8. Free energy profiles calculated from BE-META simulations of the mutant W14A in a DMPC membrane. The red and green lines correspond to the profiles obtained after 650 and 700 ns of simulation, respectively.

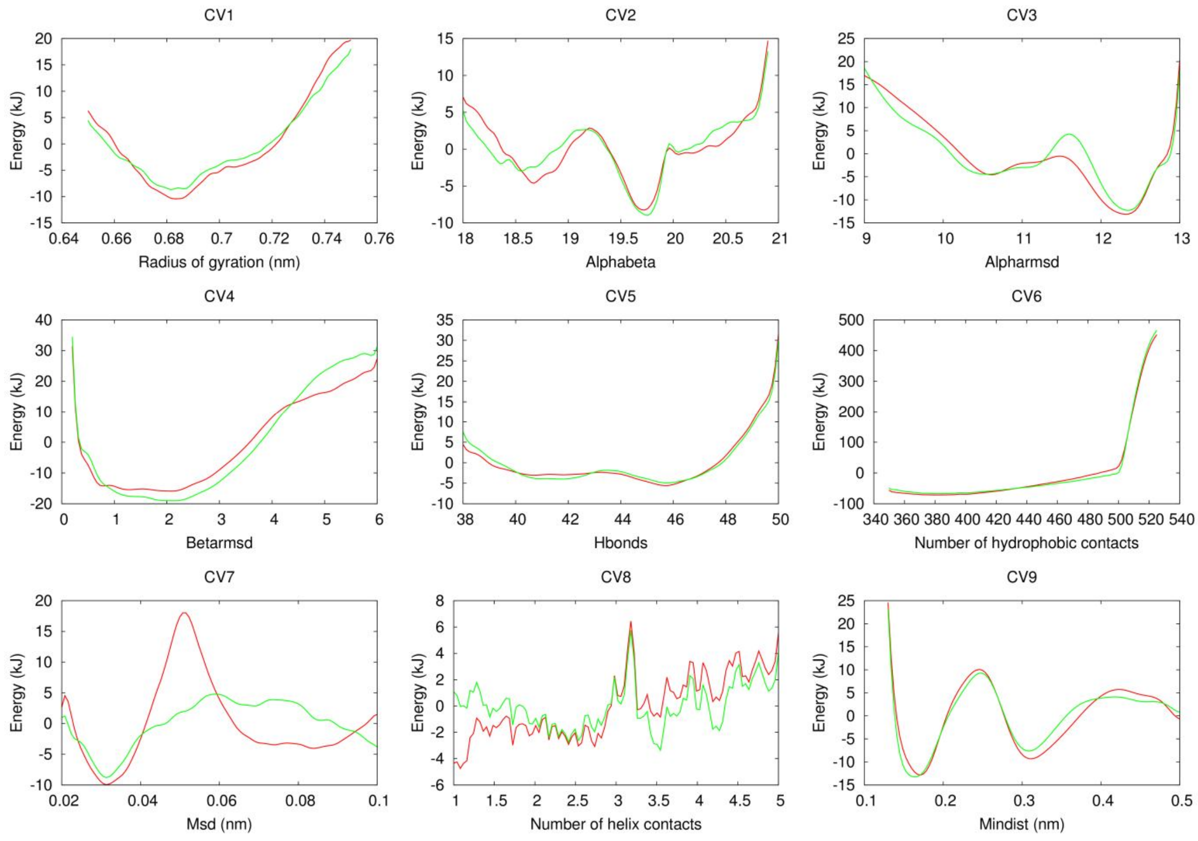

Figure S9. Free energy profiles calculated from BE-META simulations of the mutant G12A/G13A in a DMPC membrane. The red and green lines correspond to the profiles obtained after 650 and 700 ns of simulation, respectively.

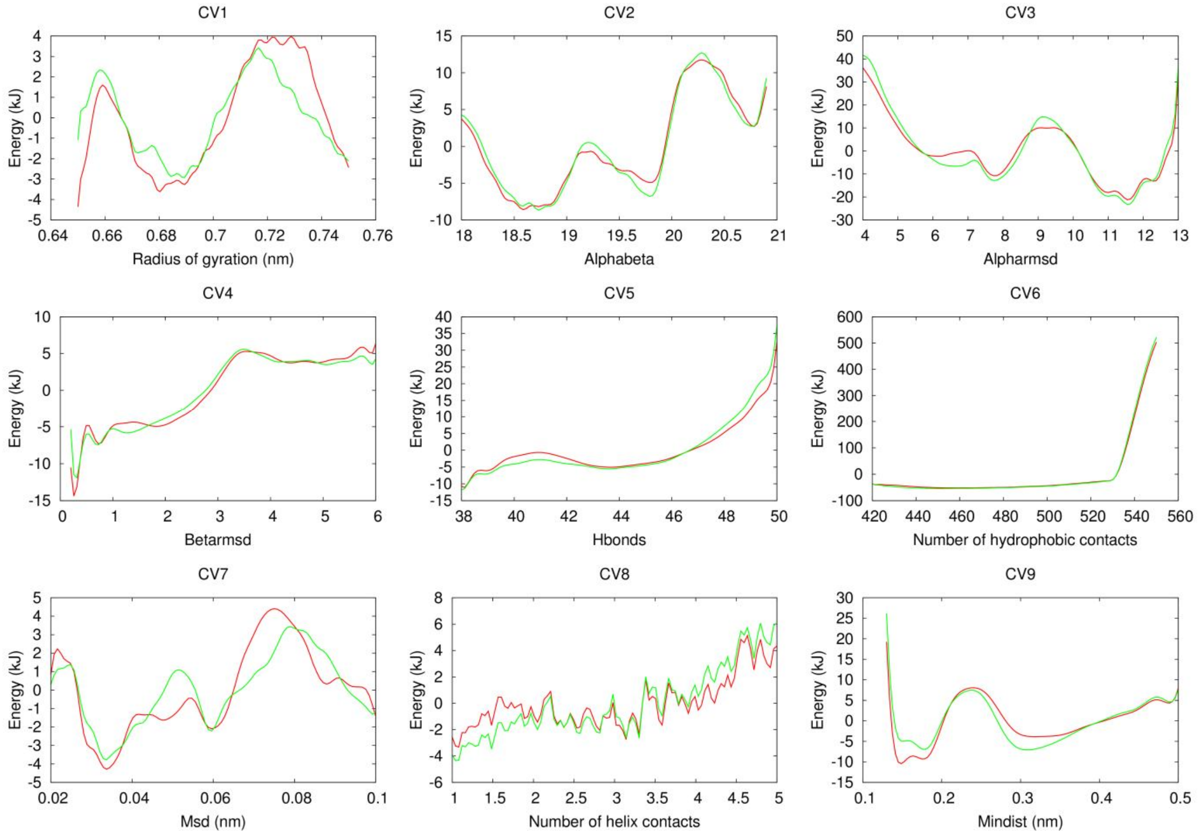

Figure S10. Free energy profiles calculated from BE-META simulations of the mutant G4A/G8A/G16A/G20A in a DMPC membrane. The red and green lines correspond to the profiles obtained after 650 and 700 ns of simulation, respectively.

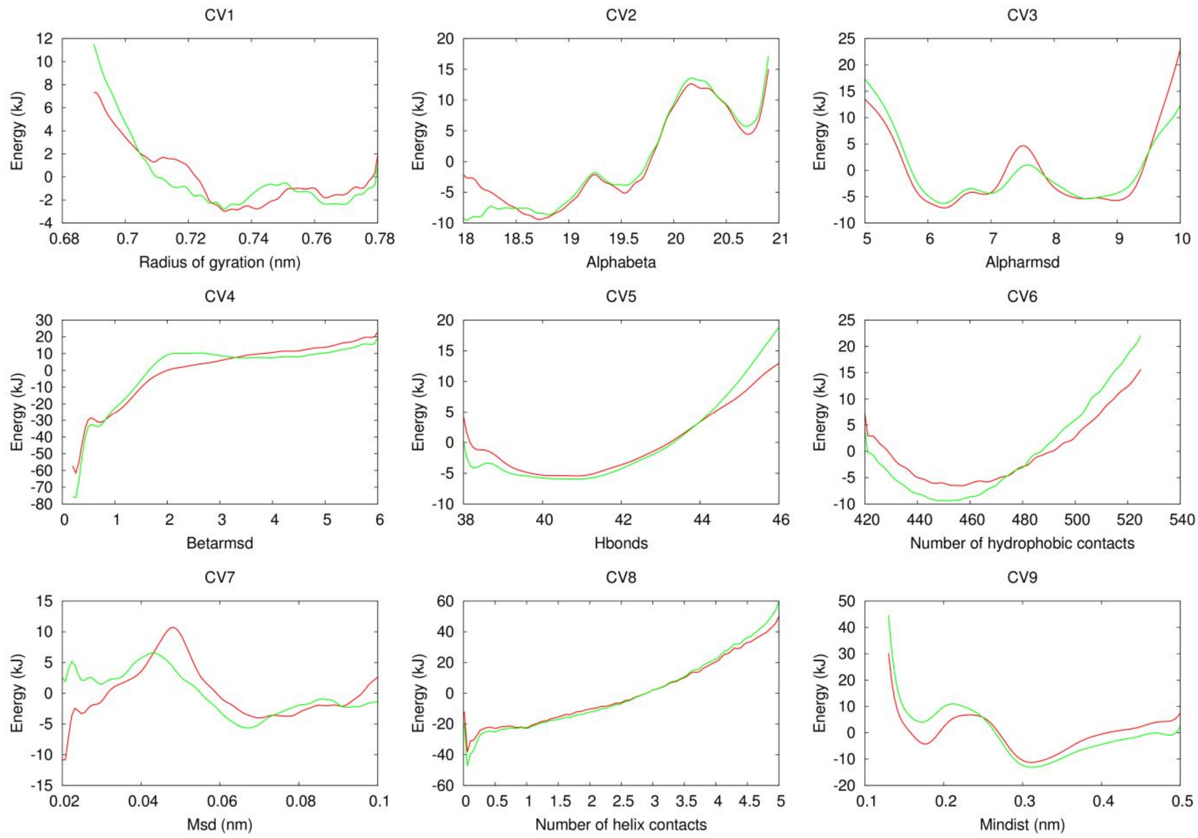

Figure S11. Comparison of the free energy profiles of the CV ALPHARMSD obtained for the WT and mutant peptides. Representative structures of each free energy minima are shown below the plots.

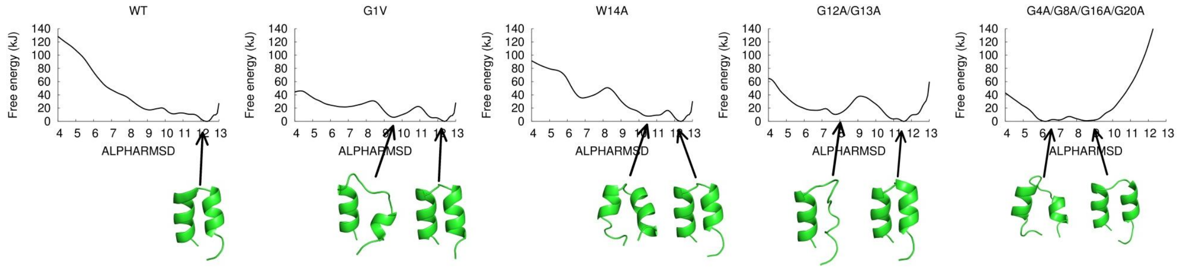

Supplement: Supplementary Information [file srep28099-s1.pdf]
